# Contusion expansion, low platelet count and bifrontal contusions are associated with worse patient outcome following traumatic brain injury—a retrospective single-center study

**DOI:** 10.1007/s00701-024-06269-7

**Published:** 2024-09-24

**Authors:** Alice S. Andersson, Iftakher Hossain, Niklas Marklund

**Affiliations:** 1 Department of Clinical Sciences Lund, Neurosurgery, Lund University and Skane University Hospital, Lund, Sweden; 2 Department of Neurosurgery, Neurocenter, Turku University Hospital and University of Turku, Turku, Finland; 3 Department of Clinical Neurosciences, Division of Academic Neurosurgery Unit, Addenbrooke’s Hospital and, University of Cambridge, Cambridge, UK

**Keywords:** Traumatic brain injury, Cerebral contusions, Bifrontal contusions, Contusion expansion, Outcome, Coagulopathy

## Abstract

**Background:**

Cortical contusions are common in moderate-severe traumatic brain injury (TBI). Cortical contusions often expand, potentially causing neuro-worsening several hours to days post-trauma. While contusion expansion (CE) may affect outcome, potential clinical and radiological markers that can predict CE have been insufficiently explored. In the present single-center retrospective observational cohort study, we evaluated clinical outcome by the Glasgow Outcome Scale extended (GOSE) scale and evaluated risk factor for CE.

**Method:**

Adult TBI patients > 18 years of age, and of all injury severities, were included. Main variables of interest were low platelet count, defined as < 150 × 10^9^/L, presence of bifrontal contusions and CE, defined as absolute contusion volume increase in cm^3^. Factors associated with CE and clinical outcome according to GOSE were analyzed.

**Results:**

Between 2012–2022, 272 patients were included. Contusion size on admission correlated positively with CE, as did the Marshall and Rotterdam radiological classification scores. Bifrontal contusions were significantly larger at admission, experienced larger CE, and had a worse outcome than contusions in other locations. Patients with a platelet count < 150 × 10^9^/L experienced a greater volume CE and had a worse outcome when compared to patients with a normal platelet count. In a multivariate analysis, CE remained significantly associated with a poor outcome six months post- injury.

**Conclusion:**

Contusion volume at admission, Marshall CT classification and Rotterdam CT score, positively correlated to CE. Bifrontal contusions and a platelet count < 150 × 10^9^/L were associated with CE, and a poor clinical outcome. Large CE volumes were associated with a worse clinical outcome, and CE was per se associated with outcome in a multivariate analysis. Management of these risk factors for CE in the acute post-injury setting may be needed to attenuate contusion expansion and to improve clinical outcome in TBI patients suffering from cortical contusion injuries.

**Supplementary Information:**

The online version contains supplementary material available at 10.1007/s00701-024-06269-7.

## Introduction

Traumatic brain injury (TBI) is a leading cause of disability and death amongst younger patients globally, and an increasing cause of mortality amongst the elderly population in high-income countries [22]. The severity of TBI is categorized into three groups based on initial Glasgow Coma Scale (GCS); severe (< 9), moderate [1, 2, 8, 13] and mild (> 12) [35]. Cerebral contusions (CC), a common type of TBI, are characterized by cortical, intraparenchymal hematomas, resulting from rapid acceleration-deceleration forces, as often seen in traumatic incidents such as motor vehicle accidents or falls [25]. The contusion forms when the intracerebral microvasculature ruptures [27]. Contusions most often occur in the basal frontal and temporal lobes where the soft brain is damaged by friction against the underlying bony skull base [25]. This makes it an injury with high risk of loss of functions associated with these areas such as personality, language, and executive functions [24]. CC are often accompanied with other intracranial hemorrhages such as epidural (EDH)-and subdural hematomas (SDH) [34].

Contusion expansion (CE), defined as the hematoma volume increases beyond its initial value, occurs in approximately half of CC cases [17, 28]. CE, visualized on a follow up computed tomography (CT) scan, is often associated with neuroworsening, *i.e.* reduced consciousness level and/or emergence of focal neurological deficits [17]. The possibility of CE makes CC challenging to manage since an awake patient presenting with minor lesions on an initial CT scan can rapidly deteriorate several hours post-injury due to increased mass effect. However, not all patient experiencing CE worsen [2, 8, 17]. The risk of CE is suggested to be the highest during the first 24 h post trauma [13]. An initial low GCS score and a larger hematoma volume at admission are associated with CE, although no consensus on the volume increase needed to define CE has been established [1]. Other suggested risk factors are male sex, old age, blood alcohol level, high international normalized ratio (INR), low platelet count and hypertension [1, 8]. A low platelet count has been associated with CE, which itself is associated with a worse long-term patient outcome [38], and trauma patients may benefit from a liberal threshold for platelet transfusion [6]. However, there are risks associated with this strategy, which should be weighed against the need for reversing antiplatelet therapy. The use of antiplatelet therapy therefore complicates CC management [32]. Intake of direct oral anticoagulants (DOAC) and, in particular, Warfarin also increases the risk for CE [5, 31].

Patients with bifrontal contusions are a common CC subgroup that tend to present with a relatively high GCS score to then rapidly deteriorate later in their clinical course due to CE [39]. This makes them prone to possible undertreatment initially, and a risk of a potentially avoidable negative outcome [10]. The optimal management of bifrontal contusions, that per se are associated with a high risk of debilitating frontal lobe dysfunction, is controversial where unilateral contusionectomy, decompressive craniectomy (DC) and conservative treatment including neurocritical care are treatment options [10, 39, 40].

In localized CC, hematoma removal via a craniotomy is indicated when a patient presents with progressive neurological deterioration and mass effect on CT, marked by significant midline shift and compression of basal cisterns, as suggested by the Marshall CT classification, and the Rotterdam CT score [21, 23]. An alternative to a contusionectomy is to perform a DC, especially in the case of diffuse cerebral swelling [7, 9]. DC has shown to lower the risk of mortality due to cerebral swelling. Although DC was associated with a higher risk of severe disability, surgically treated patients were more likely to improve over time compared to the medically treated ones. Thus, DC as a last-tier therapy for refractory intracranial hypertension may be used to reduce mortality [16, 18]. DC has also been suggested as the preferred surgical intervention for bifrontal contusions [40].

With a better understanding of the factors influencing CE and patient outcome, a more precise treatment regime can be applied to CC patients with the aim to reduce mortality and morbidity in this group of TBI patients. The aim of this study was therefore to investigate the presence of potential clinical and radiological predictors of CE. Additionally, to measure the impact of coagulopathy and presence of bifrontal contusions on CE and clinical outcome.

## Material and method

### Patient selection and data collection

**Fig. 1:**
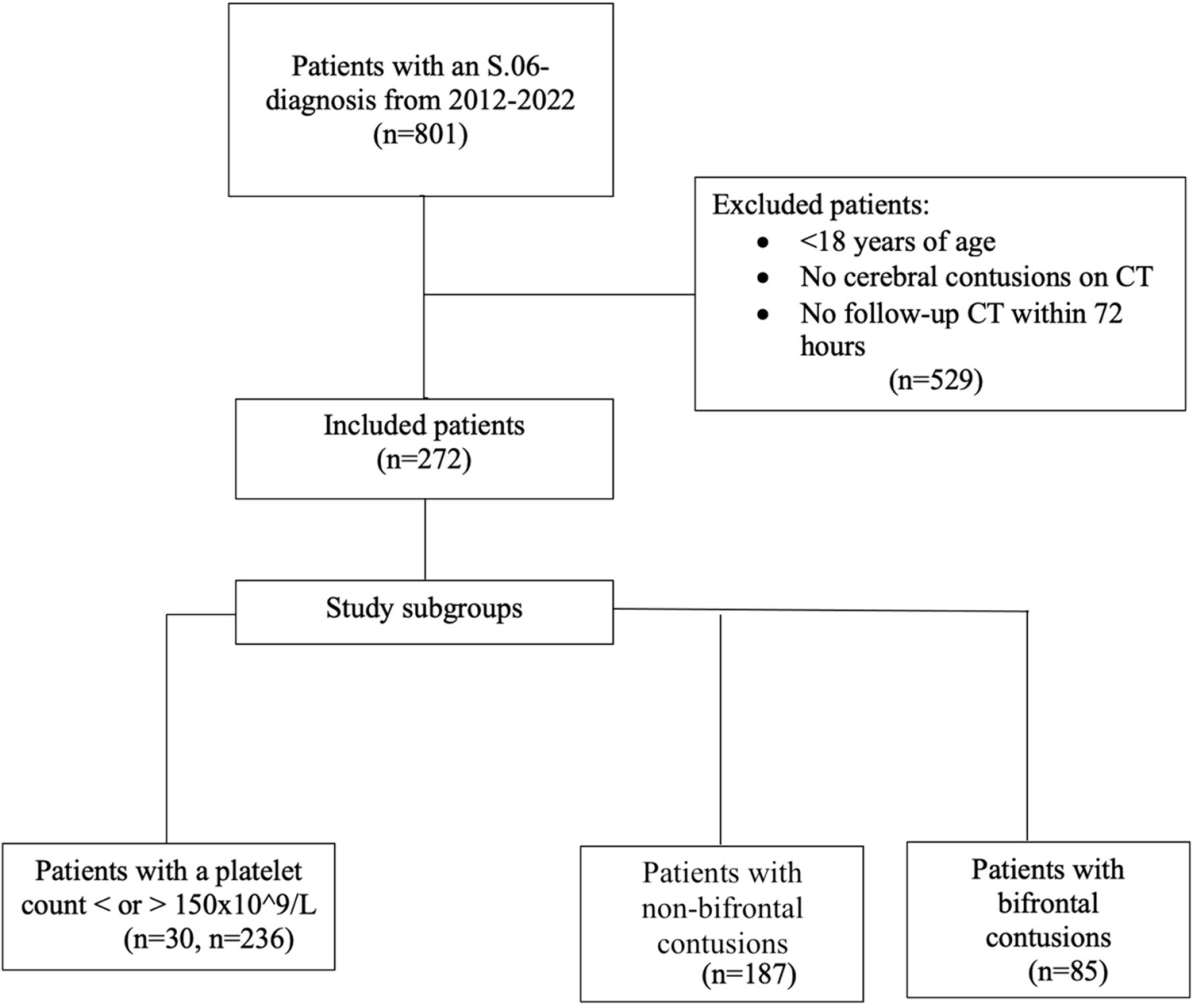
Overview of patient inclusion and subgroups. Overview of patient inclusion and subgroups

This is a retrospective study that investigates the clinical course amongst patients with CC admitted to the Neurosurgical clinic at the Skåne University hospital in Lund during the years 2012–2022. Patients who received the ICD-code S.06 (Intracranial injury) were selected to ensure all TBI patients with CC were analyzed, since CC often co-exist with other intracranial injuries. Included patients had CC present on admission CT head imaging, had a follow up CT scan within 72 h after the initial scan, and were > 18 years of age at admission (Fig. 1).

Patient information regarding age, sex, GCS, medical history, clinical-and lab parameters, treatment methods, clinical course, and outcome at six months post injury using Glasgow Outcome Scale- Extended (GOSE), were gathered from medical records accessed via Melior and SieView (Cerner, Kansas City, MO, USA). All data was stored in Microsoft Excel (Microsoft Office, Redmond, WA, USA).

We evaluated 801 patients who received a S.06 ICD-10 code between 2012–2022. After applying exclusion criteria, 272 patients were included in the study. Two subgroups were defined using contusion location: bifrontal contusions (n = 85) vs. non-bifrontal contusions (n = 187), and patients with a platelet count higher (N = 236), or lower (n = 30), than 150 × 10^9^/L blood. The majority of excluded patients were those without a contusion on CT scan.

CT = Computed tomography.

### Imaging

Radiological data was gathered from imaging archives via Sectra Picture Archiving and Communication System IDS7 (Sectra AB, Linköping, Sweden). Contusion volume was measured using the ABC/2-formula from admission and follow up CT scans of head [19]. CE was defined as the difference between the contusion volume at admission and the following CT scan performed within 72 h, and no volume cut-off was used. The largest and the second largest contusion were measured, if more than one was present, but registered separately as initial and second hematoma. The basal cisterns were registered as normal, compressed, or effaced. Midline shift was categorized as normal, < 5 mm and > 5 mm. Affected cerebral lobes, presence of bifrontal contusions, and presence of another intracranial hematoma (e.g. EDH or SDH) was registered The largest frontal contusion in bifrontal contusions was used for the comparisons between frontal and non-bifrontal contusions. Absolute contusion volume increase from first CT scan to follow up CT scan was registered, and data used in the analysis of contusion expansion. Diffuse axonal injury (DAI) was registered only if it was confirmed by Magnetic resonance imaging (MRI).

### TBI severity and outcome

The severity of TBI was classified according to GCS at admission and was registered using both the Marshall CT classification and Rotterdam CT score, using the first CT scan performed upon arrival to the emergency room [21, 23]. GOSE and GOS was used to evaluate the global functional outcome of the patients six months following TBI (Supplementary tables 1) [37]. The patient’s capacity of working, functioning independently at home, neurological deficits and capability of functioning in social relationships were extracted from the physician, physiotherapist and occupational therapist notes in the medical records six months after injury. The extracted data is corresponding to the global scale for functional outcome measurement. The data included dead to good recovery scales [37]. Extracranial injuries were was registered and Injury Severity Score (ISS) was used to asses injury severity in all patients [4].

### Coagulation profile

Blood platelet levels, information regarding INR and Activated Partial Thromboplastin Time (APTT) and platelet transfusions were collected. Antithrombotic treatment, for example intake of aspirin, clopidogrel or DOAC, was also extracted from the patient records.

### Statistical analysis

Blood platelet levels were dichotomized into two groups; low, defined as < 150 × 10^9^/L and normal, i.e. > 150 × 10^9^/L, and the patients were grouped thereafter. Blood platelet transfusion administration was considered a dichotomized variable. The largest contusion is included in the statistical analysis and for bifrontal contusions the one with the greatest volume was used. GOSE and the Glasgow Outcome Scale (GOS) was used for analysis regarding outcome, and visualized by GOS in figures [37]. When performing a multivariate analysis, the GOSE was dichotomized into two categories, unfavorable (GOSE 1–4) and favorable (GOSE 5–8), a dichotomization often used [3]. All continuous data analyzed was non-parametric, assessed by using Kolmogorov–Smirnov test, and therefore expressed in medians with interquartile range (IQ). Mann–Whitney test was used when comparing continuous data between two groups. Kruskal Wallis test was used when analyzing more than two groups. Chi-squared test was used when comparing two or more categorical variables. Spearman's rho was used for correlation analysis between continuous and/or ordinal variables. Multivariate analysis was performed using a binomial regression and is presented as odds with 95% confidence interval (CI). Level of significance was set at p < 0.05. All analyses were performed using SPSS Statistics (29.0) (IBM Corp, Armonk NY).

## Results

There were 801 patients who had an S.06-diagnosis code between the years of 2012–2022. Patients under the age of 18 years at admission and patients who had no contusions were excluded, generating 272 patients who met the inclusion criteria (Fig. 1). Of all patients, 117 (43%) had an ICP monitor inserted- 65 patients had an external ventricular drain (EVD) (24%), 32 an intraparenchymal monitor (IPM) (12%) and 20 patients (7%) had a combined intraventricular and intraparenchymatous monitor. Of all patients, 155 patients (57%) did not have any intracranial monitoring (Table 1). The median GCS score was 13 (Table 1). A majority of patients had no extracranial injuries; 191 without (70%) *vs.* 81 patients (30%) with extracranial injuries (Table 1). 35 patients underwent contusion evacuation surgery, 9 patients had a craniectomy, and three of those had both a contusion evacuation and a craniectomy procedure done (Table 1). In combination with a CC, 41 patients had an epidural hematoma and 94 patients had a subdural hematoma (Table 2). There were 31 patients who had an extra-axial hematoma evacuated, and 202 patients were conservatively treated (Table 1). The presence of extra-axial hematomas had no significant impact on outcome, χ2 (9, N = 214) = 2.814, *p* = *0.079*. The most common occurring outcome measure was GOSE 7 and 8, together representing 35% of the patient population where outcome could be assessed. GOSE was not accessible in 21% (n = 58) of patients (Table 1).

**Table 1:** Demographics of the 272 study patients

		*Median (IQ) / (n. %)*	*Missing (n. %)*
*Age*		55 (35–68)	
*Sex*	Female Male	87 (32%) 185 (68%)	
*GCS at arrival*	Mild (> 12) Moderate (9–12) Severe (< 9)	153 (56%) 49 (18%) 69 (25%)	1 (< 1%)
*Initial treatment*	Conservative Contusionectomy Craniectomy Evacuation òf extra-axial hematoma	202 (74%) 35 (13%) 9 (3%) 31 (11%)	
*Conservative to* *operative*	Continued conservative treatment Contusionectomy Craniectomy Other*	172 (85%) 21 (10%) 3 (1%) 6 (3%)	
*Cause of injury*	Fall from same level Fall from height (> 1 m) Motor vehicle accident Sports related Assault Blunt or penetrating trauma	89 (33%) 58 (21%) 83 (31%) 5 (2%) 15 (6%) 16 (6%)	
*ASA score*	1—Healthy 2—Mild systemic disease 3—Severe systemic disease 4—Systemic disease with constant threat to life 5—Moribund	133 (49%) 67 (25%) 66 (24%) 1 (< 1%) 0 (0%)	5 (2%)
*SBP admission*		140 (122–160)	17 (6%)
*Blood platelet count*		223 (183–276)	6 (2%)
*Warfarin*		11 (4%)	
*DOAC*	Dabigatran Apixaban Rivaroxoban	2 (1%) 3 (1%) 1 (0. %)	
*Antiplatelets*	Aspirin Clopidogrel Ticagrelor & Aspirin Clopidogrel & Aspirin	25 (9%) 4 (2%) 3 (1%) 3 (1%)	
*PK-INR*		1 (1–1,1)	11 (4%)
*APTT*		25 (23–27)	15 (6%)
*Days spent in NICU*		5 (2–13)	3 (1%)
*30 days mortality*		29 (11%)	4 (1%)
*Thrombosis*		10 (4%)	
*Received LMWH*		59 (22%)	
*GOSE at 6 months*	Dead or Unresponsive Severely disabled Moderately disabled Good Recovery	41 (19%) 37 (17%) 62 (29%) 74 (35%)	58 (21%)
*ICP Monitoring*	EVD IPM Combined EVD & IPM None	65 (24%) 32 (12%) 20 (7%) 155 (57%)	
*Sedative*	Propofol Midazolam Barbiturates None	57 (21%) 69 (25%) 18 (7%) 128 (47%)	
*Presence of extracranial injury/median ISS*	Yes No	81 (30%)ISS score 34 (27–41) 191 (70%) ISS score 16 (14–25)	

*GCS* Glasgow Coma Scale, *SBP* Systolic blood pressure, *PK-INR* Prothrombin complex-International ratio, *APTT* Activated partial thromboplastin time, *NICU* Neurointensive care unit, *LMWH* Low-molecular weight heparin, *ASA* American Society of Anesthesiology, *IQ* Interquartile range, *ICP* Intracranial pressure, *EVD* External ventricular drainage, *IPM* Intra-parenchymal monitor, *ISS* Injury severity score.

* = e.g. evacuation of extra-axial hematoma, or a combination of contusionectomy, evacuation of extra-axial hematoma and decompressive craniectomy

**Table 2:** Computer tomography (CT) characteristics of included patients

*Parameter (n* = *272)*		*Median (IQR) / (n. %)*
*Contusion volume at admission (largest contusion)*		6.8 ml (2.1–15.9)
*Absolute volume expansion at follow up CT scan (within 72 h)*		4 ml (4–15)
*Bifrontal contusions*		85 (31%)
*Location of largest contusion*	Frontal Temporal Parietal Occipital	150 (55%) 112 (41%) 5 (1.5%) 5 (1.5%)
*Location of second largest contusion*	None Frontal Temporal Parietal Occipital Cerebellum	133 (48%) 86 (32%) 46 (17%) 5 (18%) 1 (0.3%) 1 (0.3%)
*Epidural hematoma*		41 (15%)
*Subdural hematoma*		94 (35%)
*Basal cistern*	Open Compressed Effaced	181 (67%) 85 (31%) 6 (2%)
*Midline shift*	Normal < 5 mm shift > 5 mm shift	156 (57%) 89 (33%) 27 (10%)
*Marshall Score*		2 (2–3)
*Rotterdam Score*		3 (2–3)
*MRI verified DAI*		30 (11%)

*IQR* Interquartile range, *CT* Computed tomography, *MRI* Magnetic resonance imaging, *DAI* Diffuse agonal injury

### Contusion expansion

Of the 272 patients, 181 experienced contusion expansion (67%). The median contusion volume at admission was 6.8 ml (IQ 2.1–15.9) (Table 2). The median absolute contusion volume increase at follow-up CT scan was 4.0 ml (IQ 0–15) (Table 2). Admission contusion volume had a significant, positive correlation with absolute contusion volume increase (Spearman's rho coefficient 0.19, *p* = *0.002*). The median Marshall score was 2 (IQ 2–3) and the median Rotterdam score was 3 (IQ 2–3). Both Marshall- and Rotterdam score had a significant, positive correlation with absolute contusion volume expansion (Spearman's rho coefficient 0.26, *p* < *0.001* and 0.24, *p* < *0.001*, respectively).

There was a weak but significant correlation between absolute contusion volume increase and GCS on admission (Spearman's rho coefficient 0.14 *p* = *0.02,* and Spearman's rho coefficient -0.14 p = *0.018,* respectively). A significant difference in CE was found between the groups of "Good recovery" (1.4 ml, 0–8.2) and "Dead or Unresponsive" (14.3 ml, IQ 2–44.1), and "moderately disabled" (8.3 ml, IQ 2–18) (*p* < *0.001* and *p* = *0.001*, respectively). The presence of other intracranial hematomas did not significantly correlate to contusion expansion, nor did midline shift at admission, systolic blood pressure, known alcohol abuse, blood ethanol levels, age, or sex.

### Bifrontal contusions

The median contusion volume amongst patients with contusions at other, non-bifrontal brain regions was 4.9 ml (n = 187, IQ 1.7–13.0), and 11.6 ml amongst patients with bifrontal contusions, measuring the largest (n = 85, IQ 4.2–20,7; *p* < *0.001*). The absolute contusion volume increase amongst patients without bifrontal contusions was 2.1 ml (n = 187, IQ 0–13.2) compared to 10 ml (n = 83, IQ 2–30.6) in the bifrontal group (*p* < *0.001*). Patients with bifrontal contusions had similar GCS scores upon arrival when compared to patients with contusions in other brain regions, including patients with unilateral frontal contusions.

**Fig. 2:**
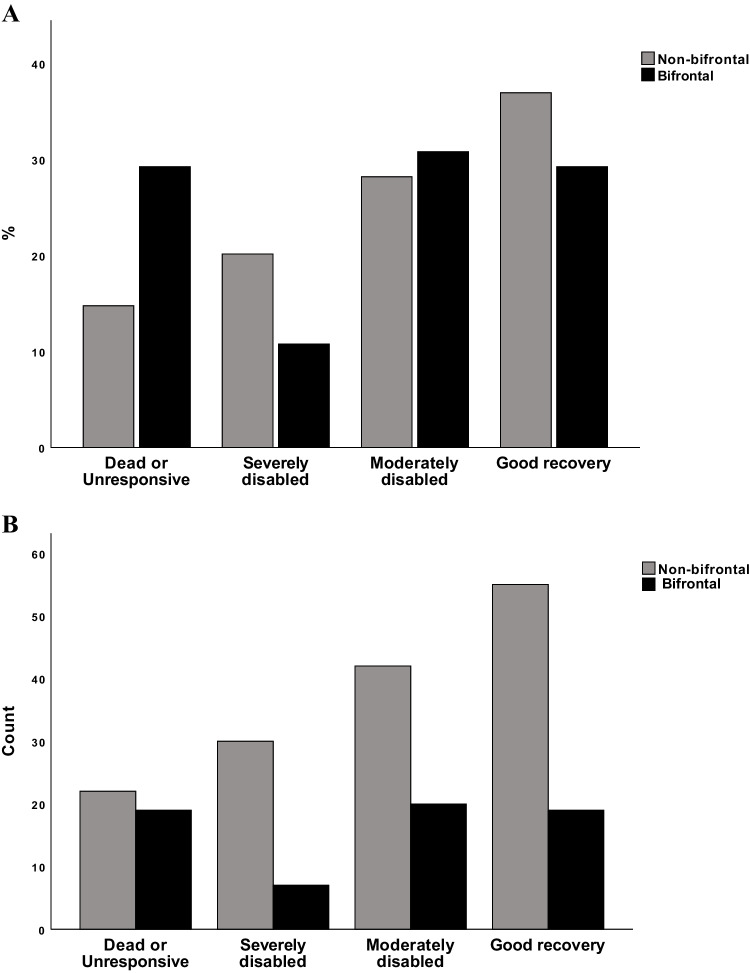
Distribution of outcome between patients with bifrontal contusions and patients with contusions in other locations. Here visualized in both percentage (A) and in count (B). Distribution between patients with bifrontal contusions (n = 65) compared to patients with contusions in other locations (n = 149) with regard to patient outcome at six months post-injury visualized using the Glasgow Outcome Scale (GOS)

Patients with bifrontal contusions did not undergo contusion evacuation surgery or craniectomy at a greater extent than other contusion patients. The median GOS in the bifrontal group was equivalent of moderately disabled (n = 65) which was also the case for the group with contusions in other locations (n = 149). Using Chi-squared test, the bifrontal group had a worse outcome than the group with other-located contusions, χ2 (3, N = 214) = 8.115, *p* = *0.0043* (Fig. 2, Table 3).

**Table 3:** Worse outcome in patients with bifrontal contusions when compared to patients with non-bifrontal contusions

Percentage of total within GOS category				
	*Dead or Unresponsive*	*Severely disabled*	*Moderately disabled*	*Good recovery*
*Bifrontal contusions (n* = *65)*	29% (n = 19)	11% (n = 7)	31% (20)	29% (n = 19)
*Non-bifrontal contusions (n* = *149)*	15% (n = 22)	20% (n = 30)	28% (n = 42)	37% (n = 55)

The difference in outcome measured as Glasgow Outcome Scale (GOS) in patients with bifrontal contusions and patients with non-bifrontal contusions are compared using Chi-square test. A total of 214 patients were included in the study of whom 65 had bifrontal contusions and 149 had contusions in other locations. The difference between the groups was significant, *p* = *0.0044*.

### Coagulation

The median blood platelet count was 223 × 10^9^/L (IQ 183–276) (Table 1). Neither INR nor APTT was significantly associated with outcome, and only 25 patients had an INR above 1.2. Median INR was 1 (IQ 1–1,1) (Table 1). For 237 patients, there were no records of any regular intake of anticoagulant drugs. There were 11 patients on Warfarin, and 25 patients were on aspirin. There was no significant difference in outcome or absolute contusion volume increase in the aspirin-treated group compared to patients not treated with aspirin. The number of patients on any other type of antithrombotic drug was too low for further analysis. Out of all patients, 11% (n = 30) received one or more blood platelet transfusions. Using Chi-square test, the patients who received blood platelet transfusion had a higher-than-expected count in the GOSE group of "dead" or "unresponsive", and none in the groups of "good recovery", in comparison to the group who did not receive a blood platelet transfusion where the trend was inverted, χ2 (3, n = 214) = 19.78, *p* < *0.001).* No significant difference in platelet transfusion could be found between patients who suffered both intra-and extracranial injuries when compared to patients with exclusively intracranial hemorrhages (*p* = *0.262*). Patients who also had extracranial injuries also did not have a significant difference in median admission platelet count when compared to patients with suffering intracranial injury (219 × 10^9^/L *vs.* 223 × 10^9^/L, *p* = *0.595*). Patients who received one or more units of platelets (n = 31) did not have a significant difference in median ISS when compared to patients who did not undergo platelet transfusion (n = 241) (20 *vs.* 25, *p* = *0.697*).

**Fig. 3:**
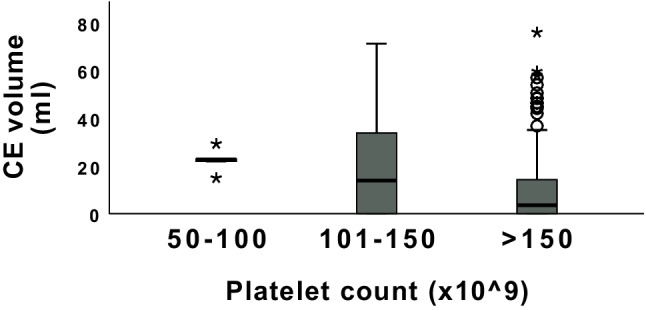
The median contusion expansion volume in patients grouped by admission platelet count. Boxplot of median and interquartile range (IQ) contusion expansion (CE) volume in patients (total n = 266) stratified into groups according to admission platelet count. Groups are 50–100 × 10^9^ (median CE volume 23 ml, n = 5), 101–150^9^ (median CE volume 14 ml, n = 31) and > 150 × 10.^9^/L, (median CE volume 4 ml, n = 230). The difference was significant (*p* = *0.005*)

**Fig. 4:**
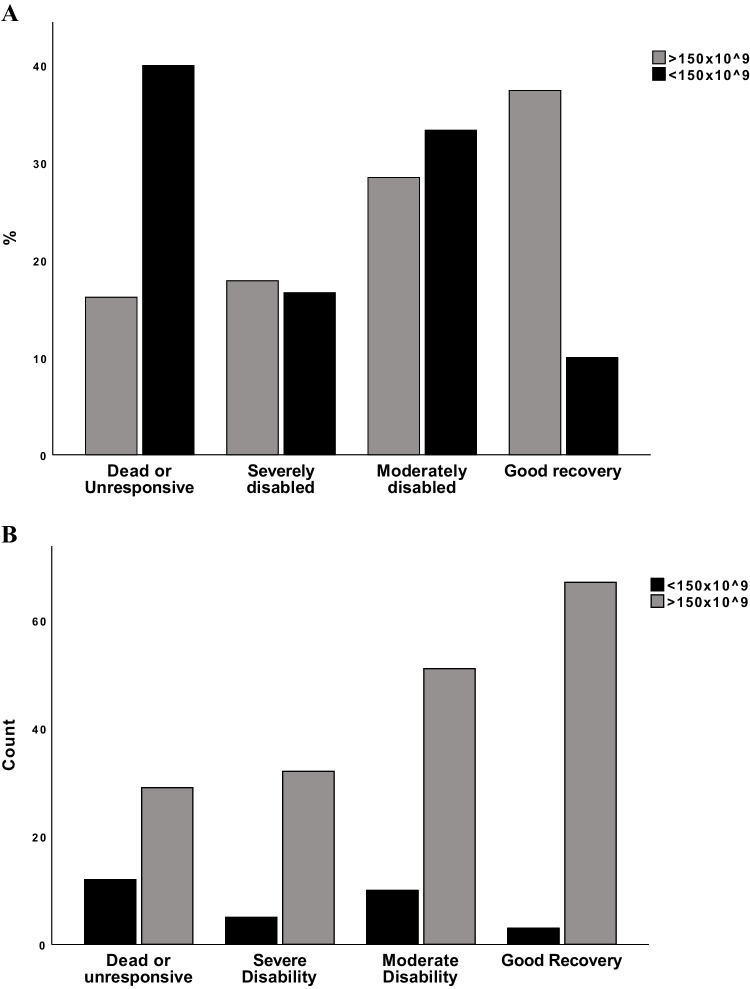
Difference in outcome between patients with a platelet count < or > than 150 × 10^9^/L. Here visualized in both percentage (A) and count (B). Distribution in patient outcome according to Glasgow Outcome Scale (GOS). dichotomizing according to platelet count less than (n = 30) or above (n = 179) 150 × 10^9^/L

When stratifying the platelet levels into three groups; 50–100 × 10^9^/L, 101–150 × 10^9^/L and > 150 × 10^9^/L, the median CE volume in each group were, respectively; 23 ml, 14 ml and 4 ml. The difference was significant (*p* = *0.005*, Fig. 3).When dichotomized to < 150- and normal (> 150 × 10^9^/L) (n = 30 and n = 179, respectively), using Chi- square test, the group with a lower platelet count had a significant worse six-month outcome according to GOSE, compared to the group with a higher blood platelet count (χ2 (3, n = 209) = 13.4, *p* < *0.004) (*Fig. 4*, *Table 4*).* A difference in absolute contusion volume increase was shown between the patients with a platelet cell count < 150 × 10^9^/L, and the > 150 × 10^9^/L group (15.3 ml versus 3.6 ml, *p* = *0.003*).

**Table 4:** The difference in outcome between patients with a low versus a normal platelet count, using Chi-square test. The difference in outcome measured in Glasgow Outcome Scale (GOS) between patients with a low platelet count versus a higher platelet count, using Chi-square test. Here, 209 patients were included, of whom 30 patients had a platelet count < 150 × 10^9^/L, and 179 had a platelet count > 150 × 10^9^/L. The difference between the groups was shown significant, *p* = *0.004*

Percentage of total within GOSE category				
	*Dead or Unresponsive*	*Severely disabled*	*Moderately disabled*	*Good recovery*
*Platelet count* < *150* × *10^*^*9*^*/L (n* = *30)*	40% (n = 12)	17% (n = 5)	33% (n = 10)	10% (n = 3)
*Platelet count* > *150* × *10^*^*9*^*/L (n* = *179)*	16% (n = 29)	18% (n = 32)	28% (n = 51)	37% (n = 67)

### Multivariate analysis

A multivariate regression was performed with six-month outcome as outcome variable. This included contusion volume at admission, absolute contusion volume difference, platelet levels < and > 150 × 10^9^/L, and whether the contusions were bifrontal or not. In the multivariate analysis, 207 patients were included (76.1%) of whom 61 patients had bifrontal contusions, and 29 patients had a platelet count < 150 × 10^9^/L. The outcome variable was dichotomized into unfavorable (n = 77) and favorable (130), as described above. The remaining variable that had a significant impact on outcome was contusion expansion (OR 0.975, CI 0.955–0.995) (Table 5).

**Table 5:** Multivariate analysis with regards to patient outcome at six months post-injury. A multivariate regression analysis was performed, including variables who on univariate regression had a significant impact (p < 0.05) on patient outcome according to Glasgow outcome scale extended (GOSE) six months post-injury. Contusion expansion was shown significantly impact outcome measured as either unfavorable (GOSE = 1–4; n = 77) or favorable (GOSE = 5–8; n = 130) outcome. Due to missing data on platelet levels upon arrival, 207 out of 272 patients could be included. OR = Odds ratio; CI = Confidence interval

*Included variables*	*n* = *207*	*OR (CI)*	*p*
*Contusion volume at admission*		0.983 (0.962–1.004)	*0.117*
*Contusion expansion*		0.975 (0.955–0.995)	***0.013***
*Contusion location (bifrontal & other)*	(61 & 146)	0.778 (0.386–1.567)	*0.482*
*Platelet levels (*< */* > *150* × *10*^*9*^*/L)*	(29 & 178)	0.490 (0.208–1.155)	*0.103*

## Discussion

In the present study, we investigated the potential clinical and radiological markers that could predict CE, and the impact of bifrontal contusions and a low platelet count on patient outcome measured by GOSE at 6-months after TBI. The Marshall CT classification and Rotterdam CT score, and a blood platelet count < 150 × 10^9^/L correlated significantly with CE. However, platelet transfusion was a negative prognostic factor for outcome measured by GOSE. Patients with bifrontal contusions had CE in a larger extent compared to the non-bifrontal group and had worse functional outcome at 6-months. CE was found as the significant variable with a negative impact on outcome 6-months post injury.

The Marshall CT classification and Rotterdam CT score previously showed good predictive accuracy for in-hospital mortality and outcome prediction in patients with TBI [29]. The Marshall CT classification has also been found to be independently associated with neuroworsening [11]. The Marshall CT classification and Rotterdam CT score, basal cistern compression and midline shift, are results of mass effect and can therefore be seen as a secondary effect of a large contusion or extra-axial hematoma. In our study, a higher Marshall CT classification and Rotterdam CT score significantly correlated with CE and CE was found to have a negative impact on 6-months functional outcome.

Coagulopathy is a complicating factor in the management of TBI patients and has been reported as a risk factor for CE in previous studies. The effect of a low platelet count has been shown to have a significant impact on CE [38]. A platelet count ≤ 100 × 10^9^/l is associated with CE and increased mortality and is usually the threshold of accepted platelet levels in elective surgery [6]. A recent Finnish multi-center intensive care study has reported that in-hospital mortality was three times higher and 12-months mortality two times higher in TBI patients with a platelet count ≤ 100 × 10^9^/l [20]. A platelet count of 150 × 10^9^/l is considered normal and was chosen as a cut-off in our study. In most previous papers, a lower platelet count was associated with CE, and our data also support this finding. Therefore, we stress the importance of rigid platelet control in TBI patients. In patients with elevated INR, double anti-platelet therapy, or any other antithrombotic treatment, the risk of CE was not significantly increased. Previously, INR levels > 1.2 were associated with a threefold increased risk for CE. However, due to the small number of patients in these subgroups in our present study, there is a risk of a type two error, and an elevated INR should be aggressively corrected [1, 36]. In our cohort, 11% received one or more blood platelet transfusions and those patients had a worse outcome when compared to patients who did not receive platelet transfusions. The indications varied widely, including as a part of a trauma transfusion protocol, and thus not merely used for a low platelet count. The pre-hospital fluid resuscitation provided to severe TBI patients may iatrogenically decrease the concentration of e.g. hemoglobin and platelet levels sampled at hospital admission, and contribute to the CE and poor outcome observed in our present study. However, we found no difference in admission ISS between patients receiving platelets when compared to patients who did not, nor in the median platelet count, between patients having extra-and intracranial injuries and patients having only intracranial hemorrhages. Since platelet transfusions are associated with a number of adverse events its role in TBI patients remains a matter of debate [26].

There is scarce literature on bifrontal contusions, and it often focuses on investigating the optimal surgical approach [10, 40]. Van de Zande et.al. performed a systematic review investigating the treatment and outcome of patients suffering bifrontal contusions [39]. Six out of seven studies of in total 356 patients measured outcome by GOS, where the average score was 4 (moderately disabled) at one-year post-injury. In the present study we observed that bifrontal contusions had a significantly larger volume when compared to contusions in other brain regions, and significantly larger CE. The median outcome was a GOSE score of 5 and 6 (i.e. moderate disability) in line with previous studies [39]. Although contusion volumes at admission were larger in the bifrontal contusion group, the GCS scores did not differ, it is plausible that patients with bifrontal contusions for anatomical reasons could accommodate a larger hematoma volume before their level of consciousness is affected. In contrast to the earlier findings, we showed that bifrontal contusions had a worse outcome when compared to other-located contusions, and thus that they may be considered an own sub-entity.

We investigated whether CE negatively impacts patient outcome, and if CE was more commonly observed in patients with a low platelet count and bifrontal contusions. Recently, a single-center retrospective study showed a 6% increased risk in a 1-point deduction on the GOS scale for every 1 ml increase of intracranial hematoma, showing a correlation between hematoma expansion and outcome at 12 months post-injury [13]. A recent multi-center observational study concluded that absolute CE outperformed relative CE in predicting both unfavorable outcome and mortality [12]**.** These results indicate that CE is a crucial contributor to outcome, consistent with our present findings where CE significantly contributed to a poor outcome both in the univariate and multivariate analysis. One feasible explanation is CE and brain swelling progression could result in increased ICP and decreased cerebral perfusion pressure (CPP), independently associated with a risk of poor outcome and death [15, 30]. One of the interesting aspects of our study is that most of the contusion cases presented as mild TBI according to the initial GCS score, but a minority had a good outcome. This was supported by a recent CENTER-TBI study that suggested the risk of initial underestimation of the severity of many TBI patients [33].

Our study is not without limitations. Firstly, no adjustments were made for the treatment intensity level, and any withdrawal of care was not registered. Secondly, the GOSE data of this study was gathered from medical journals, and 21% of the patients were lost to follow-up. Since GOSE is based on patient activity level in comparison to life pre-injury, the information was easily accessible from the medical records of patients who were in contact with medical professionals at the time for outcome evaluation. Dividing the GOSE into a dichotomous scale as favorable and unfavorable is a common division when performing multivariate analysis and is used for statistical reasons [3]. However, valuable nuances of the morbidity spectrum are lost in this simplification. Thirdly, the retrospective design of the study is another key limitation. In view of the absence of strict guidelines, the decision to admit, and to perform surgery, is mainly based on the individual neurosurgeon´s decision. Thus, there may be heterogeneity of treatments offered to the patients in our cohort.

Strengths of the study include that the study population is relatively large, and there is access to highly detailed clinical data. By defining relevant characteristics in bifrontal contusions, and in other-located contusions, we aimed to facilitate decision-making in a clinical setting. We also investigated the important role for coagulopathy for poor outcome and thus we suggest an active role for coagulation management and correction at the early stage to optimize patient outcome [14].

## Conclusion

The Marshall CT classification and Rotterdam CT score, a platelet count < 150 × 10^9^/L, contusion volume at admission and the presence of bifrontal contusions at admission correlated to CE. Patients with bifrontal contusions, a platelet count < 150 × 10^9^/L and who experienced CE suffered a worse outcome according to GOSE six months after injury, when compared to patients with contusions in other brain regions. On multivariate analysis, CE remained the most significant variable affecting patient outcome. Thus, our results support that CE is a contributing negative factor in patient outcome, and therefore a potential target for therapeutic intervention.

## Supplementary Information

Below is the link to the electronic supplementary material.

Supplementary file1 (DOCX 20 KB)

## Data Availability

Available upon request.

## Code availability

Available upon request.
