# Supplementary material for: Contusion expansion, low platelet count and bifrontal contusions are associated with worse patient outcome following traumatic brain injury—a retrospective single-center study
Source: Acta Neurochir (Wien). 2024 Sep 24;166(1):377. doi: 10.1007/s00701-024-06269-7 (PMC11422287; doi:10.1007/s00701-024-06269-7)
Supplement: Supplementary file 1 — Supplementary file1 (DOCX 20 KB) [file 701_2024_6269_MOESM1_ESM.docx]

**Supplementary table 1: Glasgow outcome scale extended (GOSE) and Glasgow Outcome Scale (GOS).**

Glasgow outcome scale extended and Glasgow outcome scale was used as a measure of patient outcome six months post-injury. Glasgow Outcome Scale was used to visualize the results in included figures.

| GOSE | Interpretation | GOS |
| --- | --- | --- |
| 1 - Dead  2 - Unresponsive | Dead  Unaware of self or environment | 1 - Dead  2 - Unresponsive |
| 3 - Lower severe disability  4 - Upper severe disability | Full assistance ADL  Partial assistance ADL | 2 - Severe disability |
| 5 - Lower moderate disability  6 - Upper moderate disability | Independent, unable to work  Partly resume work/social activities | 3 - Moderate disability |
| 7 - Lower good recovery  8 - Upper good recovery | Minor physical/mental deficit  Full recovery, or minor symptoms | 4 - Good recovery |
